# Evaluation of the primitive fraction by functional *in vitro* assays at the RNA and DNA level represents a novel tool for complementing molecular monitoring in chronic myeloid leukemia

**DOI:** 10.18632/oncotarget.24749

**Published:** 2018-04-17

**Authors:** María Sol Ruiz, María Belén Sanchez, Leandro Gutiérrez, Daniel Koile, Patricio Yankilevich, Celeste Mosqueira, Santiago Cranco, María del Rosario Custidiano, Josefina Freitas, Cecilia Foncuberta, Beatriz Moiraghi, Carolina Pavlovsky, Mariel Ana Pérez, Verónica Ventriglia, Julio Sanchez Ávalos, José Mordoh, Irene Larripa, Michele Bianchini

**Affiliations:** ^1^ Centro de Investigaciones Oncológicas-Fundación Cáncer (CIO-FUCA), Ciudad Autónoma de Buenos Aires, Argentina; ^2^ Argenomics, Ciudad Autónoma de Buenos Aires, Argentina; ^3^ Instituto de Medicina Experimental, CONICET/Academia Nacional de Medicina, Ciudad Autónoma de Buenos Aires, Argentina; ^4^ Instituto de Investigación en Biomedicina de Buenos Aires (IBioBA), CONICET, Partner Institute of the Max Planck Society, Ciudad Autónoma de Buenos Aires, Argentina; ^5^ Instituto Alexander Fleming, Ciudad Autónoma de Buenos Aires, Argentina; ^6^ Hospital Nacional Posadas, El Palomar, Buenos Aires, Argentina; ^7^ Hospital J. M. Ramos Mejía, Ciudad Autónoma de Buenos Aires, Argentina; ^8^ Fundaleu, Ciudad Autónoma de Buenos Aires, Argentina; ^9^ Hospital Interzonal General de Agudos, Prof. Dr. R. Rossi, La Plata, Buenos Aires, Argentina

**Keywords:** leukemic stem cells, chronic myeloid leukemia, tyrosine-kinase inhibitors, therapy discontinuation, persistence

## Abstract

Quantification of *BCR-ABL1* mRNA levels in peripheral blood of chronic myeloid leukemia patients is a strong indicator of response to tyrosine-kinase inhibitors (TKI) treatment. However, additional prognostic markers are needed in order to better classify patients. The hypothesis of leukemic stem cells (LSCs) heterogeneity and persistence, suggests that their functional evaluation could be of clinical interest. In this work, we assessed the primitive and progenitor fractions in patients at diagnosis and during TKI treatment using functional *in vitro* assays, defining a “functional leukemic burden” (FLB). We observed that the FLB was reduced *in vivo* in both fractions upon treatment. However, different FLB levels were observed among patients according to their response to treatment, suggesting that quantification of the FLB could complement early molecular monitoring. Given that FLB assessment is limited by *BCR-ABL1* mRNA expression levels, we developed a novel detection method of primitive cells at the DNA level, using patient-specific primers and direct nested PCR in colonies obtained from functional *in vitro* assays. We believe that this method could be useful in the context of discontinuation trials, given that it is unknown whether the persistent leukemic clone represents LSCs, able to resume the leukemia upon TKI removal.

## INTRODUCTION

Chronic myeloid leukemia (CML) is a hematopoietic disorder in which myeloid cells proliferate excessively given to the abnormal activation of the chimaeric protein BCR-ABL1. The reciprocal translocation t(9;22)(q34;q11) gives rise to the fusion gene *BCR-ABL1*, which encodes a constitutively activated protein with tyrosine kinase activity [1]. Patients are treated with specific tyrosine-kinase inhibitors (TKI), such as Imatinib, Nilotinib and Dasatinib, and the disease is monitored by measuring the level of *BCR-ABL1* transcripts in peripheral blood (PB) [2]. Despite most patients achieve a good response to TKI treatment [3], very low levels of disease can persist even after many years of successful treatment, making the decision of stopping TKI mostly restricted to clinical trials in which patients are closely monitored for rising levels of *BCR-ABL1* transcripts in PB.

Although treatment duration, deep molecular response maintenance, and Sokal score have been reported as prognostic factors of molecular relapse after TKI withdrawal [4], an explanation for the heterogeneous response after treatment discontinuation is still lacking. Molecular relapse has been attributed to the persistence of leukemic stem cells (LSCs), which are independent of BCR-ABL1 kinase activity for survival in short term *in vitro* assays [5–7]. On the other hand, a recent report suggests that their elimination *in vivo* would not be a requisite for successful TKI discontinuation [8]. However, whether the functionality of residual LSCs can vary among patients in deep molecular response remains unknown. Their potential relevance is evidenced by the great efforts that are being taken in order to develop LSCs-targeting drugs [9–11]. Therefore, the quantification and characterization of LSCs in patients under prolonged TKI treatment is of great interest in the context of assessing residual disease. This task is specially challenging in such patients because of the low frequency of LSCs in the hematopoietic system, and their low level of *BCR-ABL1* expression [12]. Therefore, new methods that allow greater sensitivity for LSCs detection are needed.

Breakpoints that lead to *BCR-ABL1* chimaeric gene are dispersed over a ∼3kbp region in *BCR* (intron 13 or intron 14), and a ∼140kbp region in *ABL1* (intron 1). Therefore, each patient harbors a specific genomic breakpoint, which requires personalized characterization in order to design patient-specific primers for PCR detection at the DNA level. Previous reports have shown that *BCR-ABL1* can be detected at the DNA level in patients under sustained deep molecular response, even in those that attained a treatment-free remission (TFR) after TKI discontinuation [13]. However, it is unknown whether the persistent *BCR-ABL1*-positive cells constitute a primitive fraction, i.e. LSCs, with the potential capacity of causing disease, or whether they represent a population of cells unable to resume the leukemia.

In order to study the dynamics of primitive and progenitor fractions, we report the evaluation of such populations at the RNA level by functional *in vitro* assays, in CML patients at diagnosis and during TKI treatment. In addition, we show that it is possible to detect residual primitive cells by *in vitro* functional assays at the DNA level, which enables independence from *BCR-ABL1* expression levels. This method is of potential interest for assessing residual disease in future TKI discontinuation trials.

## RESULTS

### Detection based on quantification of *BCR-ABL1* mRNA in primitive and progenitor fractions

**Figure 1:**
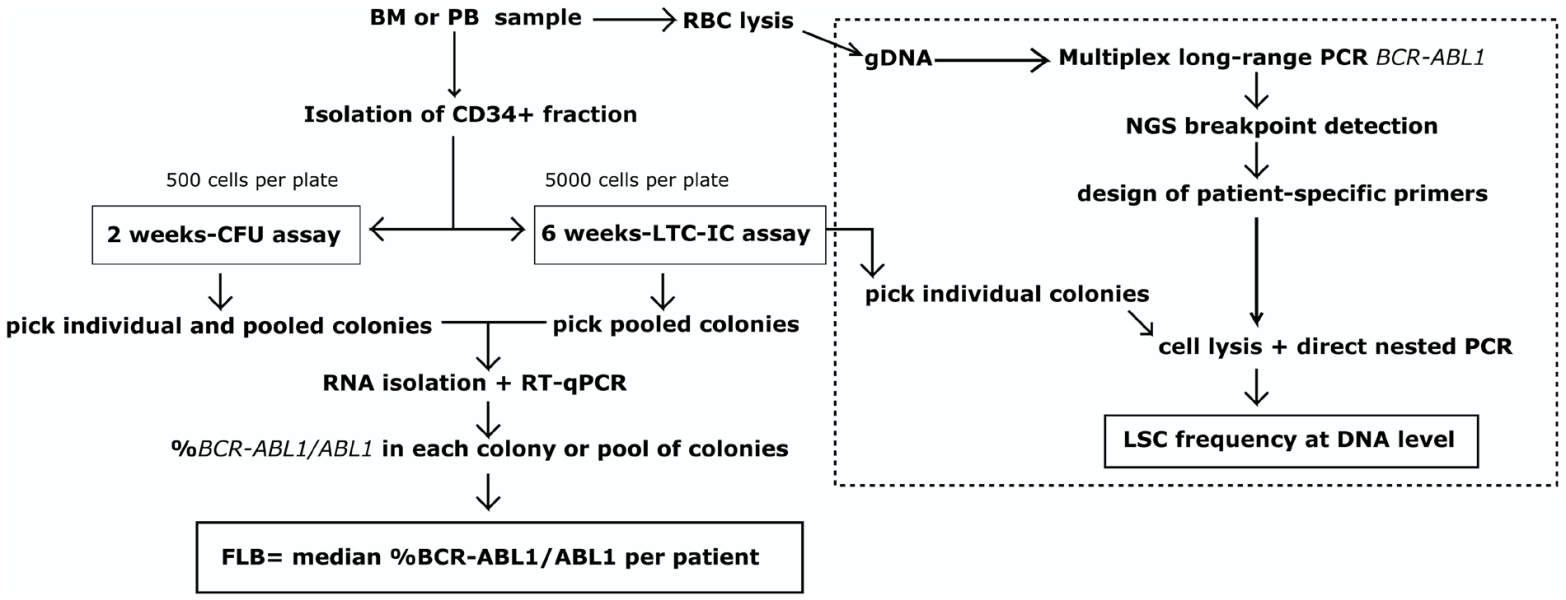
Workflow Detection of primitive and progenitor fractions at RNA level by *in vitro* functional assays was performed on patient samples at diagnosis and during TKI treatment. Evaluation of the primitive fraction at DNA level was performed as proof of concept on one patient (dotted line). BM: bone marrow, PB: peripheral blood, RBC: red blood cell lysis, gDNA: genomic DNA, FLB: functional leukemic burden, NGS: next-generation sequencing, LSC: leukemic stem cell

Long-term Culture Initiating Cell (LTC-IC) and short-term Colony Forming Units (CFU) assays are functional *in vitro* assays that enable to retrospectively quantify cells in the primitive and lineage-restricted progenitor fraction, respectively. Aiming to evaluate whether the dynamics of these populations can vary among patients at diagnosis and under TKI treatment, we performed both assays with PB or bone marrow (BM) samples from CML patients (Table 1). In order to distinguish leukemic from normal primitive or progenitor cells, we assessed the level of *BCR-ABL1* transcripts by RT-qPCR in individual or pooled colonies plucked from methylcellulose (Figure 1). Whereas initially, detection was performed on individual colonies, we observed that the evaluation of pooled colonies increased the sensitivity of the assay, allowing to scan a higher number of colonies per patient, and increasing the yield of RNA for subsequent molecular analysis.

**Table 1:** Clinical features of patients included in the study

	At diagnosis	MR^1.0^-MR^2.0^	MR^3.0^-MR^5.0^
	(N=10)	(N=13)	(N=12)
Age -yr			
Median	57	61	66
Range	18-79	36-74	28-74
Female/Male sex, %	30/70	15/85	50/50
Sokal score, no. (%)			
Low	4 (44.4) ^*^	5 (38.5)	5 (45.5) ^**^
Intermediate	3 (33.4) ^*^	4 (30.8)	5 (45.5) ^**^
High	2 (22.2) ^*^	4 (30.8)	1 (9) ^**^
TKI, no. (%)			
Imatinib	n/a	13 (100)	8 (66.7)
Nilotinib 1^st^ line	n/a	0 (0)	3 (25)
Nilotinib 2^nd^ line	n/a	0 (0)	1 (8.3)
Time of TKI treatment, months			
Median	n/a	6	20.4
Range	n/a	3-9	6-85

At diagnosis, before TKI treatment; MR- Molecular response in PB; n/a- not applicable, ^*^1 missing value, % calculated over 9 cases; ^**^ 1 missing value, % calculated over 11 cases

Given that the assessment of pooled colonies did not allow us to distinguish between the concurrently contribution of expression (i.e. *BCR-ABL1* mRNA levels) and frequency (i.e. number of leukemic cells), we considered both factors as a single one, defined as primitive or progenitor “functional leukemic burden” (FLB). For each patient, the value of FLB was calculated as the median of %*BCR-ABL1/ABL1* measured in each colony or pool of colonies (Figure 1). This definition enabled us to distinguish such effects from the previously reported “leukemic stem cell burden”, defined as the frequency of Philadelphia chromosome-positive cells (assessed by FISH) in phenotypically-defined primitive fractions sorted by flow cytometry [14, 15].

**Figure 2:**
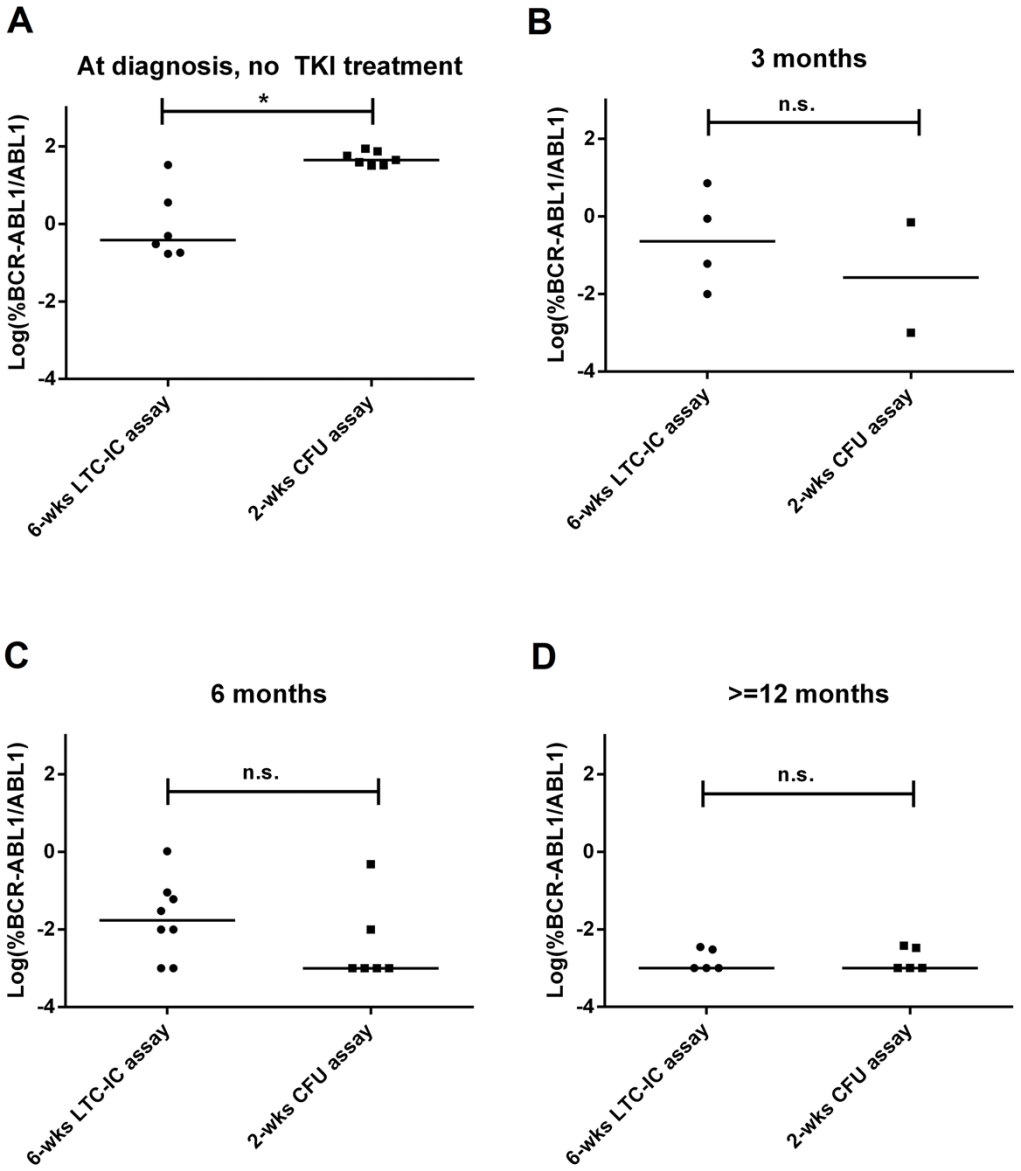
FLB in primitive and progenitor fractions at diagnosis and during TKI treatment Each dot is the median value of individual or pooled colonies from each patient: **(A)** before TKI treatment, **(B)** at 3 months, **(C)** at 6 months, and **(D)** at 12 months of TKI treatment. In order to improve visualization, data was log-transformed (zero values were assigned a value of 0.001%, according to the theoretical limit of *BCR-ABL1/ABL1* detection by RT-qPCR). Statistical analysis was performed before log-transformation. ^*^ = p<0.05, Mann–Whitney *U* test.

**Figure 3:**
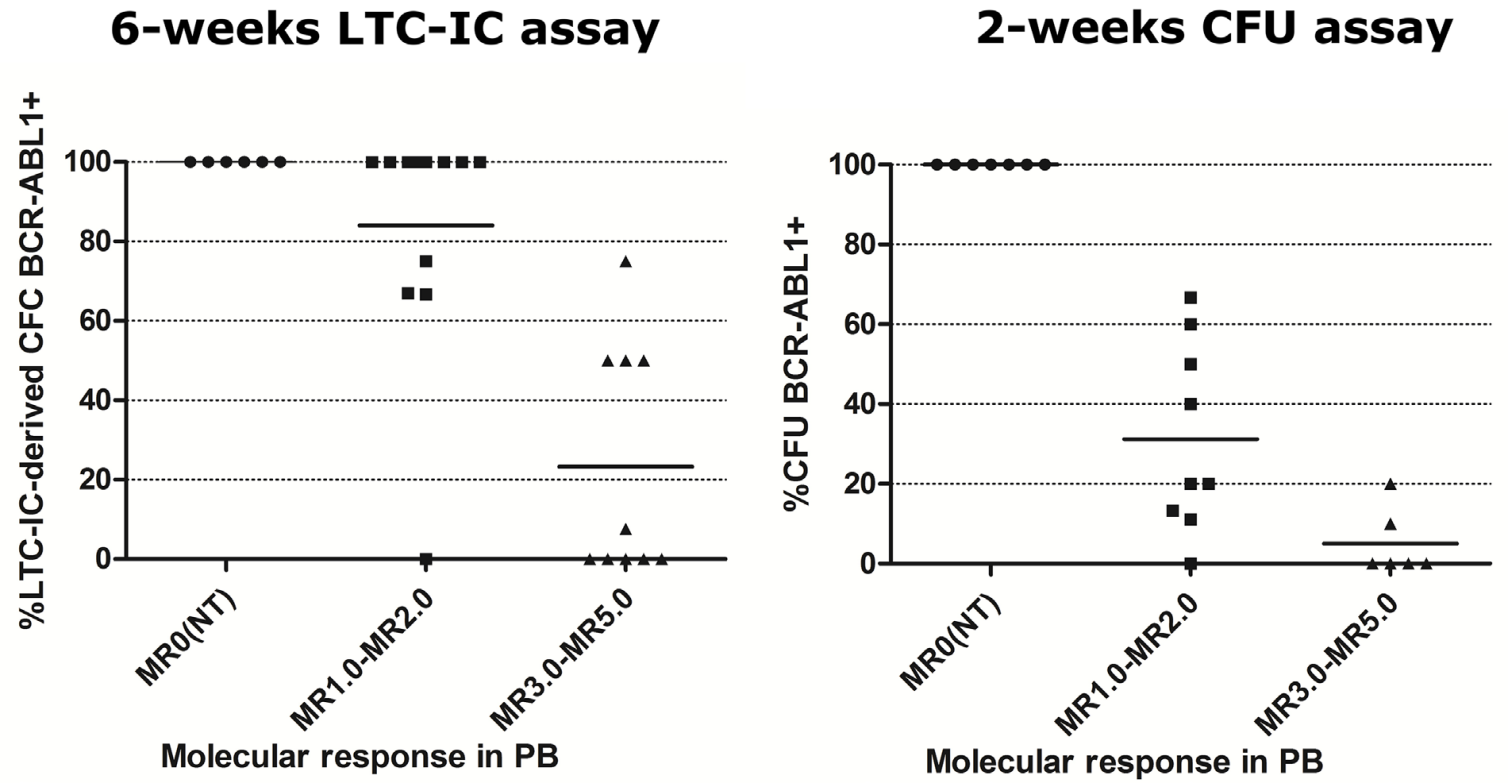
Percentage of *BCR-ABL1*+ single or pooled colonies in patients at diagnosis and under TKI treatment, according to their molecular response in PB Individual or pooled colonies were evaluated by RT-qPCR from each patient. Horizontal lines show the mean value for each group. NT= at diagnosis, before TKI treatment.

Results from patients at diagnosis before any TKI treatment showed variable levels of FLB both in the primitive and progenitor fractions, being higher in the latter (mean±SD: 6.3%±13.2% vs. 52.4%±21.5% respectively, Mann–Whitney *U* test, p<0.05, Figure 2A). As soon as patients underwent TKI treatment, the FLB was reduced both in the primitive and progenitor fractions (Figure 2B-2D). If we consider the proportion of *BCR-ABL1*^*+*^ single or pooled colonies according to the molecular response in PB, the primitive fraction showed a slower reduction than progenitor cells during treatment (Figure 3).

Early reduction of *BCR-ABL1* transcript burden in PB, such as *BCR-ABL1/ABL1*^*IS*^ ≤ 10% at 3 months, or <1% at 6 months, is associated with optimal response to first-line TKI treatment [16]. Although most of these patients can sustain an optimal response over time (No-Resistant, NR), a minority of them can develop secondary resistance (SR) to treatment. Patients that never responded to TKI treatment were considered as primary resistant cases (PR, see Materials and Methods for further details). Given that additional prognostic markers could be of clinical relevance in order to better predict resistance to TKI, we compared the primitive and progenitor FLB from both groups at different time points upon TKI treatment.

**Figure 4:**
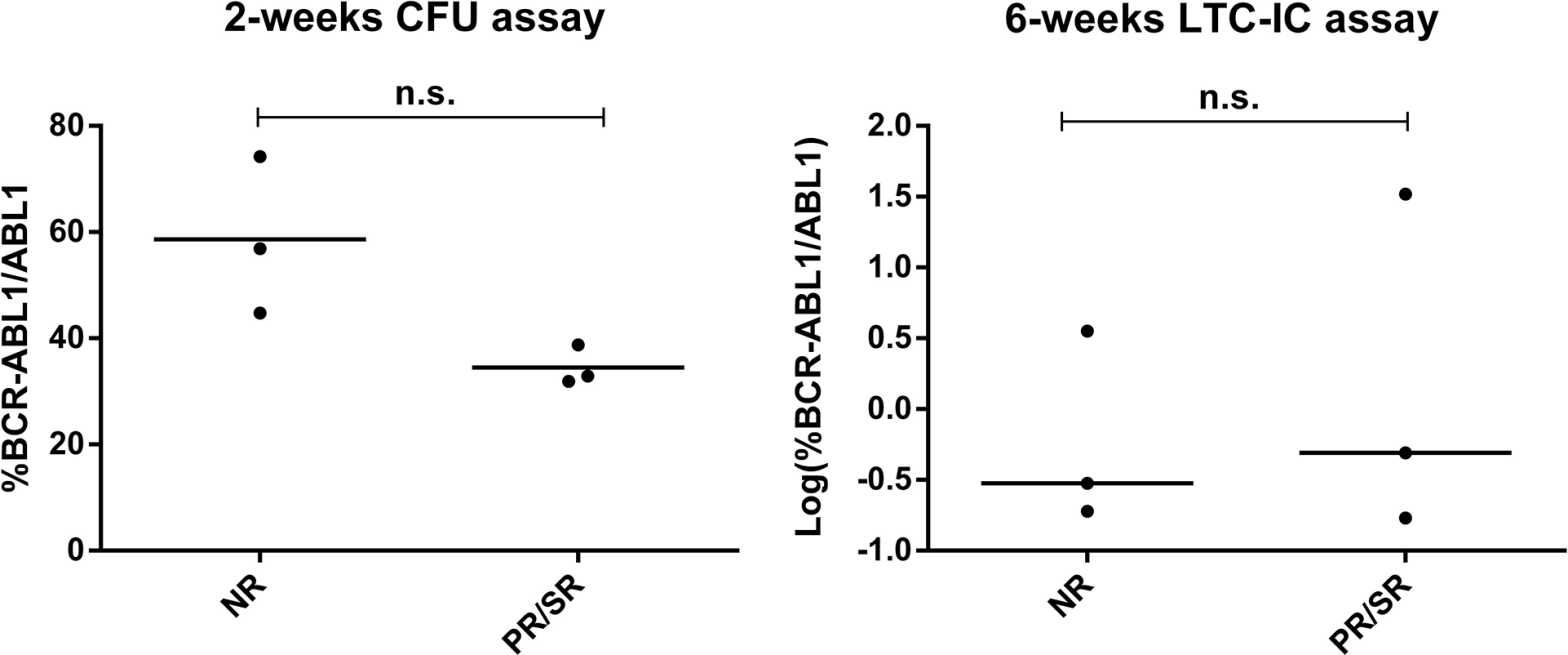
Evaluation of FLB at diagnosis %*BCR-ABL1/ABL1* was measured by RT-qPCR in individual and pooled colonies from patients at diagnosis, before any TKI treatment, in the progenitor (left) and primitive (right) fractions. Each dot is the median value of individual or pooled colonies from each patient. In order to improve visualization, data from 6-wks-LTC-IC assay was Log-transformed. Statistical analysis was performed before log-transformation, n.s: p>0.05, Mann–Whitney *U* test.

**Figure 5:**
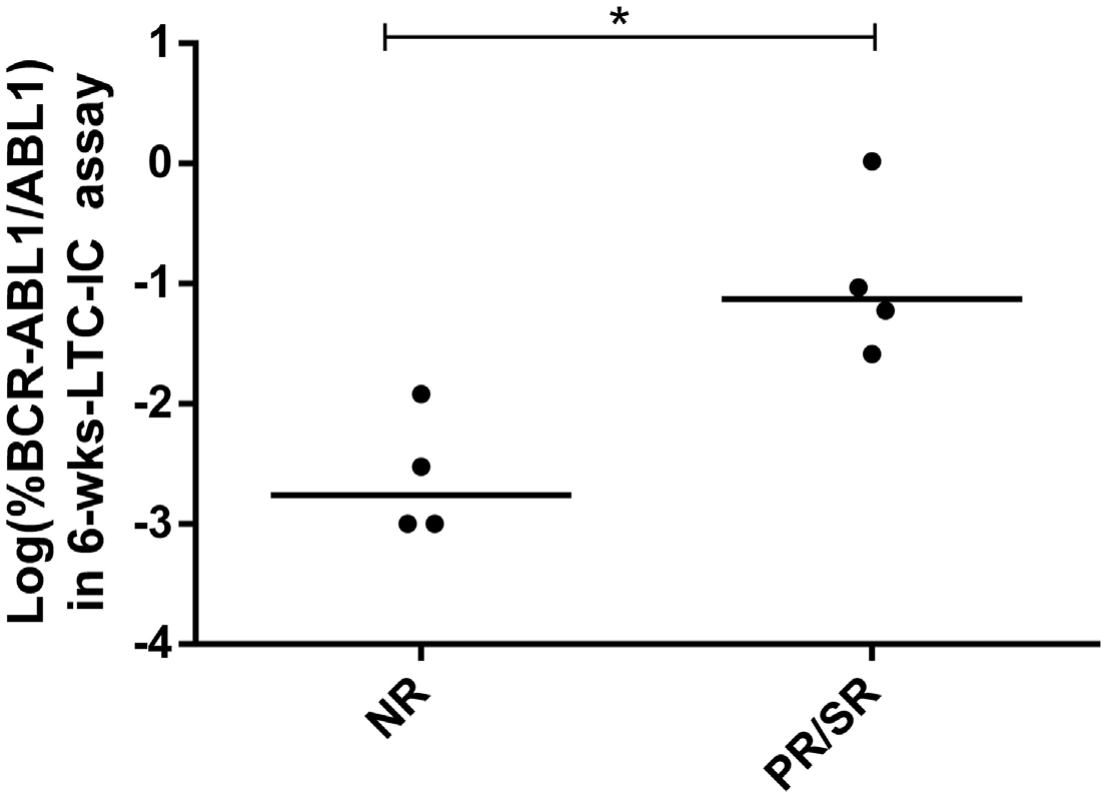
Primitive FLB in patients at 6 months of TKI treatment *%BCR-ABL1/ABL1* was measured by RT-qPCR in colonies from 6-wks-LTC-IC assays. Each dot is the median value of pooled colonies from each patient. In order to improve visualization, data was log-transformed (zero values were assigned a value of 0.001%, according to the theoretical limit of *BCR-ABL1/ABL1* detection by RT-qPCR). Statistical analysis was performed before log-transformation. N=4 per group, ^*^ = p<0.05, Mann–Whitney *U* test.

Despite the fact that at diagnosis we did not observe significant differences neither in the progenitor nor in the primitive fraction between patients in the NR and PR/SR groups (Figure 4), at 6 months of TKI treatment patients in the PR/SR group showed significantly higher levels of primitive FLB than those in the NR group (p<0.05, Mann–Whitney *U* test, Figure 5).

### Detection based on amplification of *BCR-ABL1* DNA in the primitive fraction

**Figure 6:**
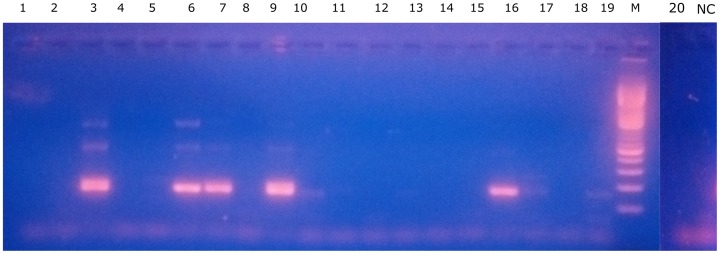
Patient-specific *BCR-ABL1* gDNA detection in the primitive fraction 2.5% agarose gel electrophoresis result from nested PCR using patient-specific primers. Estimated size of 2nd-round specific PCR product: 166bp. Lanes 1-20: individual CFU-Cs, NC= negative control (surrounding methylcellulose with no CFU-Cs), M: 100-bp molecular weight marker. Lanes 3, 6, 7, 9 and 16 show BCR-ABL1^+^ 2nd-round-PCR product.

Regarding the possibility of assessing residual disease in patients showing deep molecular response, we were not able to detect *BCR-ABL1* transcripts in most patients with the LTC-IC assay (Table 2). Given that detection is limited by the level of expression of *BCR-ABL1* mRNA by individual colonies, we designed patient-specific primers in order to evaluate *BCR-ABL1* rearrangement at the DNA level. A sample of PB at diagnosis was used for genomic DNA (gDNA) extraction and sequencing of the rearrangement breakpoint by long-range multiplex-PCR followed by next-generation sequencing (NGS) (Supplementary Figure 1). The patient showed declining levels of *BCR-ABL1* transcripts in PB under Imatinib treatment (400mg daily), attaining a MR^3.0^ at 18 months. A PB sample at 18 months of TKI treatment was used for CD34^+^ cells isolation, followed by a 6-weeks-LTC-IC assay (Figure 1). We assessed the presence of *BCR-ABL1*-positive individual colonies by an in-house method of direct nested-PCR, without a gDNA extraction step (see Materials and methods). Quality of the starting material was evaluated by detecting an independent gene at the DNA level, and colonies from a 2-wks-CFU assay at 6 months of treatment were used as positive controls. Nested PCR showed improved sensitivity compared to single PCR (data not shown), and it was able to detect a low proportion of *BCR-ABL1*^*+*^ individual colonies in the primitive fraction (5 out of 20) (Figure 6).

**Table 2:** Percentage of patients with at least one pool of colonies positive for *BCR-ABL1* mRNA

Molecular response in PB	6-weeks LTC-IC assay
MR^0^ (No treatment)	100% (6/6)
MR^1.0^-MR^3.0^	88% (15/17)
MR^4.0^-MR^5.0^	20% (1/5)

## DISCUSSION

The assessment of individual LTC-IC-derived cells is particularly challenging: first, LSCs can be highly quiescent and, therefore, might not generate any detectable progeny in these assays [5]. Second, this cell population, being in the apex of the hematopoietic system, is of very low frequency, emphasizing the methodological difficulty in detecting these cells in BM or PB samples. In designing the present study, we first sought a method that would allow the functional isolation from a CD34^+^ cell population of a viable fraction of stem-like leukemic cells. For this, we used the most rigorous *in vitro* LSC detection technique (6-weeks LTC-IC) to evaluate the effects of TKI therapies on the most primitive leukemic cell compartment. As stated above, we considered the FLB to reflect a population of primitive or progenitor cells able to self-renew, give rise to daughter cells during *in vitro* functional assays, and produce variable levels of *BCR-ABL1* mRNA. We had to adopt this definition because the evaluation of pooled colonies did not allow us to distinguish the effects of variable frequency of *BCR-ABL1*-positive colonies, from the effects of variable levels of *BCR-ABL1* expression among individual colonies. Despite this limitation, dynamics of the primitive fraction during TKI treatment measured by FLB was comparable to previous reports based on phenotypically defined LSCs [14, 15], complementing such studies. As far as we are concerned, this is the first time that the primitive fraction is measured by LTC-IC assays in patients during their first year of TKI treatment.

Previous reports suggested that *BCR-ABL1* overexpression in primitive cells (lin^-^CD34^+^CD38^-^) and mature (CD34^+^CD38^+^) progenitors may contribute to the failure of TKIs to prevent disease progression [17]. However, disease behavior is very variable for individual patients, with some progressing within a few months, and others remaining in stable CP for many years. This heterogeneity may relate to mutations later acquired in the *BCR-ABL1* gene, subtype of stem cell in which *BCR-ABL1* is expressed, differences in transcript expression patterns between patients, and differential interactions with the BM microenvironment and the immune system [18–23]. Here we show that, at six months of treatment, the primitive FLB in CP CML patients could be a prognostic marker correlating with resistance to TKIs. Although a larger cohort of patients should be studied in order to confirm our observations, these results reinforce previously reported data about the impact of LSC burden on TKI therapy outcome [14, 15], adding new evidence based on functional *in vitro* assays.

The fact that longer treatment is associated with lower relapse rates upon TKI interruption suggests that the risk of relapse might be related to the achievement of certain depth of MR below the detection limit of conventional RT-qPCR [24]. In multiple TKI-discontinuation trials, around 60% of patients relapse, whereas 40% can maintain TFR [4]. It has been shown that patients that achieve TFR can harbor *BCR-ABL1*-positive clones assessed at the DNA level [13], or at the RNA level [8]. However, it is not known whether such persistent cells are potentially clonogenic, i.e. LSCs that are able to self-renew and give rise to a full leukemic clone, or whether they are terminally differentiated cells that could survive TKI treatment by some unknown mechanism. A third possibility is that the immune system in TFR patients allows the presence of LSCs that are not able to develop their leukemic potential [25]. For this reason, strategies that increase PCR sensitivity for detection of residual LSCs may be relevant to better select patients with the highest likelihoods of remaining in remission without treatment. Although detectable levels of *BCR-ABL1* mRNA in individual colonies from patients with undetectable deep molecular response have been reported, such levels were very low [26], suggesting that RNA-based methods will always be limited in patients under TKI treatment for long periods of time. In this work, we describe a strategy that, combining direct-PCR assessment for BCR*-ABL1* DNA breakpoint with LTC-IC assay for separation of the primitive hematopoietic fraction, enables us to measure residual leukemic disease at the LSC level. Therefore, this method could be particularly useful in situations where the concurrently low burden of leukemic primitive cells and very low *BCR-ABL1* mRNA expression levels, limit the sensitive detection of residual disease. With the purpose to validate this strategy, we will apply it in a currently recruiting TKI discontinuation trial in Argentina; we believe that in the future, this study may result of great interest in order to understand patients´ heterogeneous capacity of successfully maintaining TFR.

Overall, our results suggest that assessment of *BCR-ABL1* mRNA levels in the primitive fraction during TKI treatment could be of clinical utility in order to improve and complement molecular monitoring in PB. Finally, we propose that the residual leukemic burden should be further assessed by gDNA PCR at the LSC level.

## MATERIALS AND METHODS

### Patient samples

The project was approved by the Institutional Review Board, at Instituto Alexander Fleming, Buenos Aires, Argentina. After giving written informed consent, BM or PB samples were obtained from newly diagnosed, untreated CML patients in CP, or from patients at different time points during TKI treatment. Mononuclear cells were isolated by density-gradient centrifugation (Ficoll-Paque PLUS, GE Healthcare Life Sciences) for 30 minutes at 480xg, followed by one wash in PBS, a red cell lysis step (EDTA 0.13mM, KHCO_3_ 1 mM, NH_4_Cl 170mM, pH 7.3), and a low-speed centrifugation step (12-15 minutes at 200xg) for removal of the platelet-rich fraction. Up to 2x10^8^ mononuclear cells were used for CD34^+^ cells isolation. Patients were classified according to their response to TKI treatment as follows: patients that were not able to achieve early response milestones and that would be considered as cases of warning or treatment failure according to the European Leukemia Net [16], were classified as cases of PR to treatment; patients that initially responded to treatment, and met early response (3 and 6 months) milestones, but later developed intolerance, rising levels of *BCR-ABL1* transcripts, or secondary malignancies, were considered as SR patients; patients that sustained the requirements to be considered as optimal responders up to 1 year of TKI treatment, and did not develop SR events were considered as NR patients.

### CD34^+^ cells isolation

In order to enrich samples in the progenitor and primitive fraction, we performed a positive selection using CD34 MicroBeads (Miltenyi Biotech), according to the manufacturer’s instructions. The CD34^+^ fraction was immediately used for functional assays, or cryopreserved for later use in freezing medium (DMEM 50%/HSA 40%/DMSO 10%). When available, 10,000 cells were used for purity assessment by flow cytometry.

### CFU assay

500 CD34^+^ cells were plated in duplicate in p35 culture dishes containing 1mL of enriched methylcellulose (Methocult H4435 Medium, Stem Cell Technologies, Vancouver, Canada), and incubated at 37°C in a humid chamber. After 14-18 days, hematopoietic colonies were counted under the microscope, and individual or pooled colonies (made of 4 to 6 colonies) were plucked from methylcellulose and put into 500μl of RPMI medium (GIBCO, Thermo Fisher Scientific). Up to 10 pools of colonies were evaluated per patient, depending on the yield of the assay. All types of colonies were used for RNA isolation (CFU-GM, CFU-M, CFU-G, BFU-E, CFU-GEMM), and no differences in *BCR-ABL1* mRNA expression were found among different types (data not shown). After centrifugation, pellets were resuspended in 100μl of lysis solution (RNAqueous-Micro Kit, Ambion, Thermo Fisher Scientific), and kept at -20°C until RNA extraction was performed.

### LTC-IC assay

5,000 CD34^+^ cells were plated in collagen-coated p35 culture dishes containing 2mL of Long-term culture medium (Myelocult supplemented with freshly prepared hydrocortisone 10^-6^ M, both from Stem Cell Technologies), over a feeder layer composed of murine M2-10B4 and SI/SI cells producing hIL-3, hSCF and hGCSF (kindly provided by Stem Cell Technologies), previously irradiated at 80Gy. Transfected M2-10B4 and SI/SI cells were selected with G418 and Hygromicin B every 2 or 3 passages. One half of the medium was replaced every week. After 6 weeks, adherent and non-adherent cells were harvested, and 20,000 cells were used for short-term CFU assays, as described above.

### RNA extraction and RT-qPCR

Total RNA was extracted from single or pooled colonies using RNAqueous-Microkit (Ambion, Thermo Fisher Scientific). RNA was eluted in 18μl of elution solution at 75°C; only samples with acceptable purity (A_260_/A_280_ ≥ 1.7) were used for retrotranscription using Superscript II (Invitrogen, Thermo Fisher Scientific). qPCR was performed using primers and probes for *BCR-ABL1* and *ABL1* from the European against cancer program [27]. Absolute quantification was performed using a calibration curve from a commercial kit (Molecular MD). Total RNA from PB samples was extracted using Trizol (Invitrogen, Thermo Fisher Scientific) after lysis of red blood cells. Results were harmonized to the International Scale by means of a conversion factor obtained by standardization with secondary cellular calibrators [28]. Some patients were monitored in other standardized centers, and so *BCR-ABL1/ABL1* ratios were obtained directly from their data.

### NGS detection of *BCR-ABL1* breakpoint

High quality genomic DNA was extracted from PB or BM samples from newly diagnosed CML patients using DNAzol (Invitrogen, Thermo Fisher Scientific). Multiplex long-range PCR was performed using different sets of primers as previously described [13]. The PCR product was used for library generation in the Ion Torrent PGM platform (Thermo Fisher Scientific) according to manufacturer’s instructions. Briefly, PCR products were fragmented with Ion Shear Plus Reagents and purified with Agencourt AMPure XP (Beckman Coulter). Barcoded adapters were ligated according to manufacturer’s instructions with Ion Xpress Plus Fragment Library Kit, and the products were quantified by qPCR. Finally, samples were prepared for emulsion PCR, enriched with Ion PGM Template OT2 200 Kit, and sequenced using Ion 318TM Chip v2 with Ion PGM Sequencing 200 Kit v2.

### Bioinformatics analysis of NGS data

In order to find the chromosomal translocation point, reads were aligned using the Subjunc aligner included in the Subread package (v1.5.0-p3) [29] and human genome reference UCSC version of the hg19. Sorting and indexing were performed with Picard (v1.119) (http://broadinstitute.github.io/picard). The fusion points were localized using CREST (Clipping Reveals Structure, v0.0.1) [30], which relays on soft-clipping signatures for identifying breakpoints. Finally, Blast2Seq algorithm was used against *ABL1* and *BCR* reference sequences in order to identify each segment.

### Detection of *BCR-ABL1*^+^ cells at DNA level using NGS-derived data

Sequence corresponding to patient-specific fusion breakpoint was used for PCR primer design (Primer3Plus). Short and long-term culture assays were performed as described above. Individual colonies were plucked from methylcellulose and subjected to direct nested PCR without a DNA extraction step. For this, cell lysis was performed by plucking individual colonies with a p20 tip set at 5μl, into 50 μL of the following mixture: GoTaqFlexi PCR buffer (Promega, USA) supplemented with NP-40 0.5%, Tween-20 0.5%, and proteinase K 0.91 mg/mL. Lysates were incubated for 1 hour at 60°C, followed by 15 minutes at 95°C. 10% of the final volume was used for first-round PCR. This PCR product was diluted ten-fold for second-round PCR.

### Statistical analysis

Data was plotted using GraphPad Prism software or R project. Statistical analysis of the data was performed with Infostat Software (Córdoba, Argentina). When the assumptions of normality and homoscedasticity of the data were not met, variance was modeled previous to performing a parametric test (ANOVA), or analyzed by non-parametric tests (Mann–Whitney *U* test). A mixed-effects model was used in ANOVA (response to treatment as fixed-effect type, patient as a random-effect type).

## SUPPLEMENTARY MATERIALS FIGURE



## Abbreviations

CML: chronic myeloid leukemia
TKI: tyrosine-kinase inhibitors
PB: peripheral blood
BM: bone marrow
RT-qPCR: quantitative reverse transcription polymerase chain reaction
LSC: leukemic stem cells
TFR: treatment-free remission
LTC-IC: Long-term Culture Initiating Cell
CFU: Colony Forming Units
FLB: functional leukemic burden
PR: primary resistance
SR: secondary resistance
NR: no resistance
gDNA: genomic DNA
NGS: next-generation sequencing
CP: chronic phase
MR: molecular response
