# Supplementary material for: Evaluation of the primitive fraction by functional in vitro assays at the RNA and DNA level represents a novel tool for complementing molecular monitoring in chronic myeloid leukemia
Source: Oncotarget. 2018 Apr 17;9(29):20255–64. doi: 10.18632/oncotarget.24749 (PMC5945542; doi:10.18632/oncotarget.24749)
Supplement: Supplementary file 1 [file oncotarget-09-20255-s001.pdf]

Evaluation of the primitive fraction by functional *in vitro* assays at the RNA and DNA level represents a novel tool for complementing molecular monitoring in chronic myeloid leukemia

SUPPLEMENTARY MATERIALS

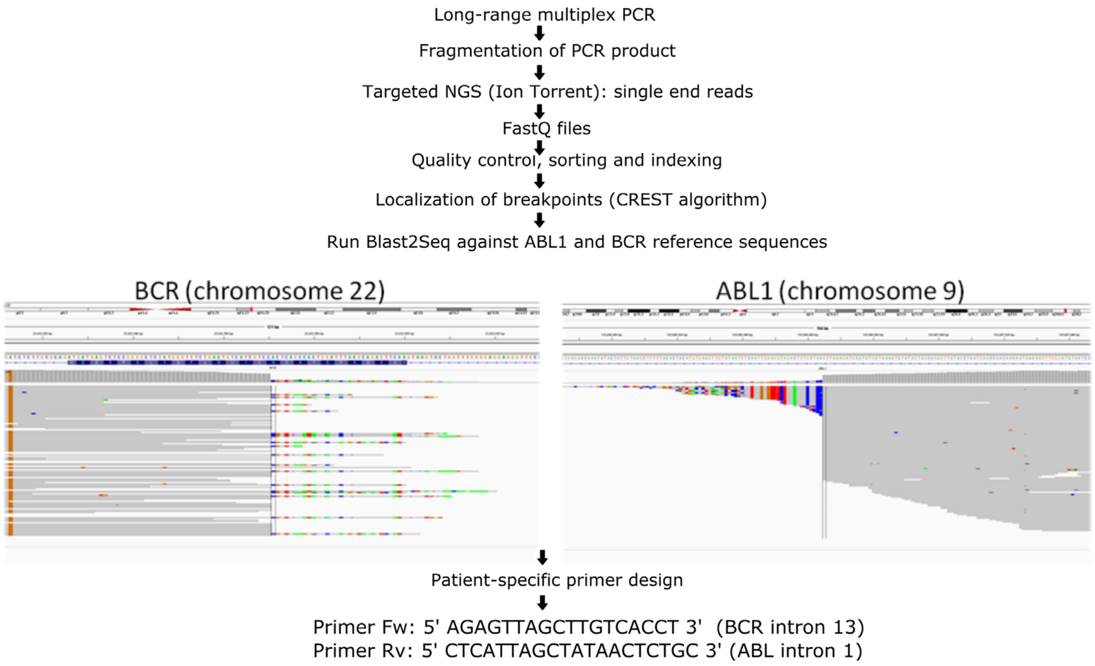

Supplementary Figure 1: *BCR-ABL1* breakpoint detection. gDNA from a sample at diagnosis was used for amplification of *BCR-ABL1* breakpoint region by long-range multiplex PCR. PCR products were sequenced by NGS in order to find the breakpoint sequence. A scheme of the bioinformatics pipeline used for detection of breakpoint regions from single-end reads is shown; the detailed pipeline is available in the section "Materials and Methods". The consensus sequence was used for the design of patient-specific primers in order to detect *BCR-ABL1*-positive cells by PCR in the primitive fraction from the same patient under TKI treatment.
